# Bound states in the continuum: From fundamental physics to emerging photonic paradigms

**DOI:** 10.1016/j.isci.2026.114803

**Published:** 2026-01-29

**Authors:** Shubin Zhang, Ye Fan, Yufei Ma, Meixue Zong, Handong Sun, Zhiqiang Yang, Zhengji Xu

**Affiliations:** 1 School of Microelectronics Science and Technology, Sun Yat-sen University, Zhuhai 519082, China; 2 Guangdong Provincial Key Laboratory of Optoelectronic Information Processing Chips and Systems, Sun Yat-sen University, Zhuhai 519082, China; 3 Institute of Applied Physics and Materials Engineering, University of Macau, Macau, China

**Keywords:** Physics, Photonics, Applied sciences

## Abstract

Bound states in the continuum (BICs) defy the conventional intuition of open photonic systems by supporting perfectly localized eigenmodes within the radiative spectrum. Originally conceived in quantum mechanics and later realized in photonics, BICs have evolved from a theoretical curiosity into a unifying framework for engineering high-*Q* resonances in periodic optical structures. Unlike conventional high-*Q* modes that rely on fine parameter tuning to suppress radiation, BICs arise from symmetry enforcement, destructive interference, or momentum-space topology, endowing them with intrinsic robustness and distinct design principles. This review provides a cohesive perspective on the physical origin, theoretical foundations, and emerging functionalities of BICs in photonic crystal slabs, metasurfaces, and related platforms. By bridging band theory, temporal coupled-mode theory, and multipole analysis with experimentally accessible observables, we elucidate how BIC physics enables rational control of confinement, radiation, and modal coherence. We further highlight recent advances in quasi-BIC platforms, demonstrating how controlled radiative coupling facilitates enhanced emission, nonlinear processes, and non-local wavefront manipulation. Looking forward, the integration of BIC concepts with topology-assisted design and reconfigurable photonic architectures points toward scalable, multifunctional, and intelligent photonic technologies.

## Graphical abstract

**Figure:**
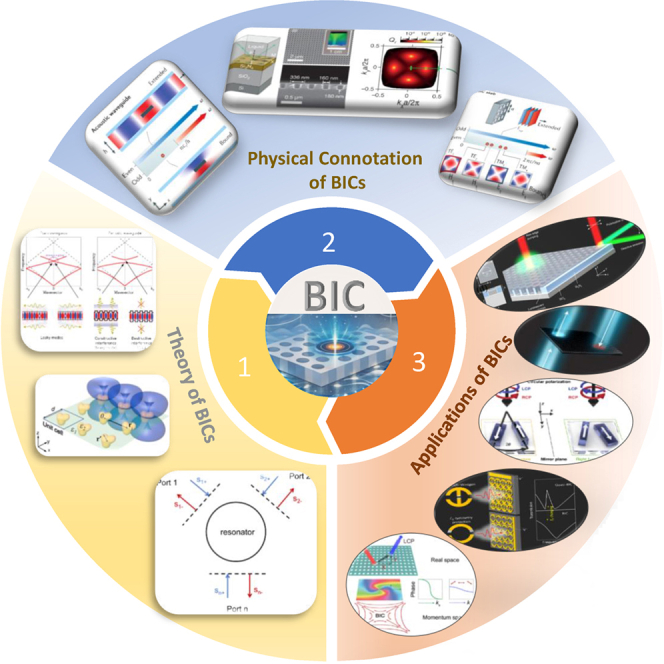

## Introduction

With the rapid development of optics and materials science, periodic optical structures—such as photonic crystal slabs and metasurfaces—have emerged as crucial research directions in the fields of micro-nano photonics and integrated optics, owing to their capability of precisely manipulating electromagnetic fields at the subwavelength scale and their unique light-matter interaction mechanisms.^1^^,^^2^^,^^3^^,^^4^ The introduction of BICs (bound states in the continuum) has recently led to breakthroughs in this field. As a special type of electromagnetic eigenstate whose frequency lies within the radiative continuum yet exhibit strong spatial confinement, BICs possess a theoretically infinite *Q*-factor (quality factor), exotic polarization properties, and a compact mode volume. These features offering a new route to high-performance photonic devices.

Although BICs are often discussed alongside high-*Q* resonances in open photonic systems, it is crucial to distinguish their physical origins and practical implications. In photonic crystal slabs and metasurfaces, high-*Q* resonances can indeed be achieved through careful geometric tuning that suppresses radiative leakage. From a phenomenological viewpoint, such resonances may resemble BICs when their linewidths become extremely narrow. However, their underlying mechanisms are fundamentally different. Conventional high-*Q* resonances correspond to local optima in parameter space and rely on fine-tuning, making them intrinsically sensitive to fabrication imperfections and environmental perturbations. In contrast, ideal BICs arise from symmetry enforcement or topological constraints that forbid radiation by principle rather than by optimization. As a result, perturbations typically displace BICs in parameter or momentum space instead of eliminating them, endowing BIC-based platforms with a form of robustness inaccessible to conventional resonators.

The topological nature of many photonic BICs further elevates their significance beyond that of tunable high-*Q* modes. The association of BICs with polarization vortices and quantized topological charges in momentum space implies that their existence and evolution are governed by global conservation laws. This perspective enables deterministic creation, migration, and annihilation of BICs through well-defined topological processes, and provides systematic design strategies based on symmetry breaking, topological charge engineering, and merged-BIC configurations. These features position BICs not merely as another route to high-*Q* resonances, but as a fundamentally different paradigm for controlling light in open systems.

In terms of material systems, all-dielectric structures have become an ideal platform for high-performance BIC devices, owing to their low intrinsic loss, CMOS compatibility, and ease of integration.^5^^,^^6^^,^^7^ Through rational structural design, BIC modes can be excited in all-dielectric metasurfaces, which effectively suppresses radiative loss and achieves high-*Q* resonance. This provides key technical support for applications including optical sensing, optical communication, nonlinear optics, and bio-optics.

Currently, the design and fabrication of high-*Q* all-dielectric metasurfaces remain key challenges in this field. Systematic research is needed on physical mechanisms, *Q*-factor enhancement, structural optimization, and fabrication processes. Furthermore, it is essential to expand the application prospects of BICs in interdisciplinary fields including integrated optics, topological photonics, information processing, and bio-optics.

BICs possess compact mode volumes, which originate from the highly localized nature of their intrinsic field distributions. Owing to the suppression of radiative losses in BIC modes through destructive interference or symmetry-protection mechanisms, their electromagnetic energy is strongly confined within subwavelength-scale structural units. This characteristic endows BICs with unique advantages in applications requiring strong field enhancement and miniaturized integration.

Against this backdrop, BICs—as a type of electromagnetic eigenstate that possesses frequencies within the radiative continuum yet exhibit complete energy localization—have become a critical topic in cutting-edge optical research. Their core value lies in providing a physical basis for the realization of devices, such as ultra-high-*Q* resonators, ultra-sensitive sensors, and narrowband filters. Although ideal BICs (with infinitely large *Q*-factors) are difficult to achieve in experiments, q-BICs can be constructed through structural modulation, thereby significantly enhancing device performance.^8^^,^^9^^,^^10^^,^^11^^,^^12^ In nonlinear optics, BIC-based microcavities confine light to deep-subwavelength volumes, dramatically enhancing light-matter interactions and thus boosting second-harmonic generation, four-wave mixing, and other nonlinear processes.^13^^,^^14^^,^^15^

However, the practical application of BICs still faces numerous challenges. First, BICs are extremely sensitive to processing precision. Nanoscale deviations can cause a pronounced *Q*-factor drop, placing stringent demands on processing technologies. Second, although multiband BICs have been theoretically predicted, experimental verification, and independent modulation methods are still underdeveloped. Furthermore, q-BICs are typically accessed via symmetry breaking, which makes them relatively sensitive to incident angles and polarization and thus limits their applications. Meanwhile, BICs correspond to exceptional points in momentum space, and their topological properties, when coupled with dissipative channels, can give rise to novel physical phenomena. However, research on reconfigurable BICs in non-adiabatic systems remains largely unexplored. Therefore, overcoming the technical bottlenecks and fully tapping into the application potential of BICs will be a key direction for future research.

While several excellent reviews have summarized the formation mechanisms and application platforms of BICs, the present work aims to provide a distinct and integrative perspective. Rather than organizing BICs solely by symmetry protection, interference conditions, or application categories, we emphasize BICs as a unifying physical framework that connects momentum-space topology, global modal coherence, and multifunctional photonic control. By highlighting the roles of topological charges, polarization singularities, and non-local modal responses, this review bridges disparate research directions, such as non-local wavefront modulation, polarization singularity engineering, quasi-BIC-enabled enhancement, and reconfigurable photonic devices.

Moreover, this review places particular emphasis on translating abstract theoretical models into system-level design principles. By explicitly linking band theory, temporal coupled-mode theory, and multipole analysis to practical performance metrics—including bandwidth, robustness, and energy efficiency—we illustrate how BIC physics supports rational and scalable photonic design beyond empirical optimization. Through critical comparison with conventional high-*Q* resonances and identification of emerging opportunities enabled by topology and non-local effects, this work constructs a coherent through-line that positions BICs as a foundational platform for next-generation flat optics, topological photonics, and reconfigurable light-matter interaction systems.

The remainder of this review is organized as follows. Overview of bound states in the continuum introduces the fundamental concepts and classification of BICs, providing a unified physical picture of symmetry-protected and accidental BICs in periodic photonic systems. Fundamental theory of BICs summarizes the core theoretical frameworks, including band theory, temporal coupled-mode theory, and multipole analysis, and clarifies their relevance to practical device design. Cutting-edge applications of BICs in periodic optical systems reviews recent advances in BIC-enabled applications, with particular emphasis on emerging frontiers such as non-local wavefront modulation and momentum-space polarization singularity control. The first part of conclusion presents a critical and forward-looking discussion of challenges and opportunities in materials, architectures, fabrication tolerance, and system-level integration. Finally, conclusion concludes the review with a summary and outlook.

## Overview of bound states in the continuum

### Physical connotation and implementation mechanisms of BICs

BICs are a special class of physical states that exist in both quantum mechanics and classical wave systems (e.g., photonics, acoustics). Their core characteristic is as follows: although the frequency or energy of this eigenstate lies within the continuous radiative spectrum of the system—where, in theory, energy leakage should occur due to coupling with radiative modes—the eigenstate can achieve complete localization without any radiation.

The concept of BICs can be traced back to the theoretical investigation of quantum mechanical potential scattering problems by von Neumann and Wigner in 1929. By explicit mathematical construction, they showed that spatially oscillating potentials of a specific form allow the Schrödinger equation to support eigenstates that combine bound-state localization with continuum-frequency character.^16^ However, due to the extremely strict requirements of early theoretical models on the form of the potential function, BICs remained difficult to realize and observe experimentally for a long time. In 1985, Friedrich and Wintgen published two seminal studies in Physical Review A, which were based on multichannel quantum defect theory and Feshbach resonance theory, respectively. These works revealed that BICs can be formed through the interference of different resonances in coupled Coulomb channels. Taking hydrogen atoms in a uniform magnetic field as a prototype for verification and further extending the findings via model calculations, the authors clarified the universal mechanism underlying BICs, thereby laying a crucial foundation for the development of this field.^17^^,^^18^ In recent years, with the development of artificially microstructured materials (such as photonic crystals and metasurfaces), BICs have not only been experimentally verified in optical systems but also become one of the important physical mechanisms for manipulating optical fields and enhancing light-matter interactions.

Unlike the idealized configurations that are difficult to realize in quantum systems, BICs in classical wave systems allow for precise control over their existence and parameters through the design of artificial structures, making them much easier to observe and study experimentally. Since the early 21st century, BICs have been widely reported in classical wave systems (e.g., electromagnetic waves, acoustic waves) and quickly emerged as a cutting-edge research focus. With the maturation of fabrication technologies for artificial micro-nano structures such as photonic crystals and metasurfaces, research on BICs has achieved significant progress in optical systems. In 2008, Marinica, Borisov, and Shabanov were the first to introduce the concept of BICs into optical grating systems.^19^ In the same year, Bulgakov and Sadreev pioneered the extension of this concept to optical waveguide systems. By constructing a photonic crystal waveguide model with a defect layer and deriving a non-Hermitian effective Hamiltonian, they confirmed that optical BICs could be realized by adjusting the structural parameters of defects.^20^ In 2011, Plotnik’s group experimentally observed BICs in coupled waveguide arrays, verifying the crucial role of structural symmetry in suppressing radiation and laying an experimental foundation for subsequent studies on BICs in systems such as photonic crystals.^21^ Since then, research on BICs has expanded from the original quantum systems to a wide range of classical wave domains, covering multiple systems including electromagnetic waves, water waves, acoustic waves, and elastic waves.^22^^,^^23^^,^^24^^,^^25^^,^^26^^,^^27^^,^^28^^,^^29^^,^^30^

**Figure 1:**
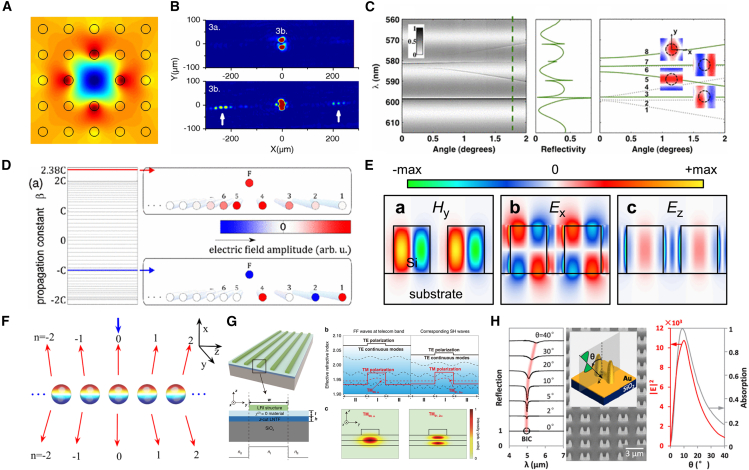
Schematic overview of representative research on optical BICs across different photonic platforms (A–D) BIC phenomena realized in fiber-based systems. (E–G) BIC-related effects in planar waveguides and photonic crystal structures. (H) BICs in metasurface systems.^19^^,^^20^^,^^21^^,^^31^^,^^32^^,^^33^^,^^34^^,^^35^^,^^36^^,^^37^^,^^38^^,^^39^^,^^40^^,^^41^^,^^42^^,^^43^ Copyright American Physical Society, Springer Nature and American Chemical Society.

Within the scope of optics, BICs are understood as a special type of optical eigenstate: although their frequencies lie within the radiative continuum—where, in theory, they should couple with radiative modes and result in energy leakage—they can actually achieve complete spatial localization without radiating to the far field. The physical mechanism underlying this counterintuitive phenomenon is that certain specific symmetries, destructive interference, or parameter optimization cut off the coupling between BICs and all radiative channels. This enables BICs to exhibit non-radiative properties similar to those of bound states in closed systems, even in open environments. Currently, optical BICs have been extensively realized and studied in various dielectric structures, providing new insights for the design of high-performance photonic devices. Research on optical BICs has been carried out in waveguide, fiber, photonic crystal, and metasurface systems, as shown in Figure 1.^19^^,^^20^^,^^21^^,^^31^^,^^32^^,^^33^^,^^34^^,^^35^^,^^36^^,^^37^^,^^38^^,^^39^^,^^40^^,^^41^^,^^42^^,^^43^

**Figure 2:**
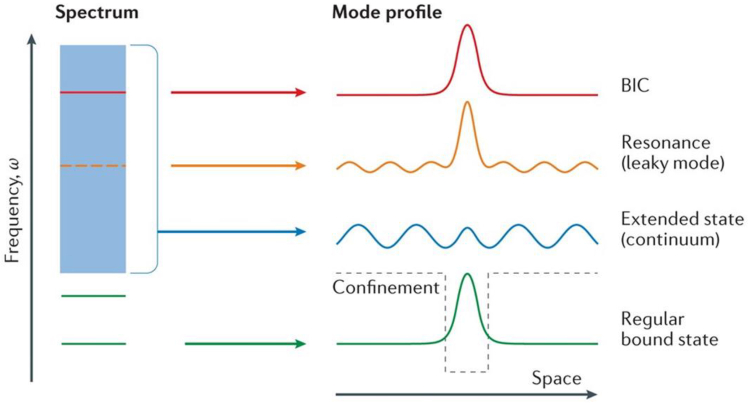
Definition of BIC Reproduced from ref.^44^ with permission from Springer Nature (2016).

As illustrated in Figure 2, consider an open optical system, the fundamental frequency spectrum of the photonic system consists of one or more radiative states of the continuous spectrum (in blue) and discrete bound states that do not radiate outward (in green). The spatial localization of bound states is induced by structural or potential confinements (black dashed lines). Resonant modes (in orange) typically emerge as finite radiative states, resulting from the coupling between bound states within the continuous spectrum and radiative waves. BICs (in red) are special states that lie inside the continuum but remain localized without radiation. From the perspective of wave spectrum structure, the eigenfrequency of BICs differs from that of conventional bound states: the latter are usually located in the bandgap region below the radiation threshold, whereas the eigenfrequency of BICs falls within the radiative light cone or continuous spectrum. In this scenario, the eigenstates of the system should theoretically be leaky states or scattering states. However, due to the orthogonality between the wavefunction and radiative modes in terms of symmetry, interference phase, or spatial distribution, the coupling amplitude is completely canceled out. This fully suppresses the outward leakage of energy, forming a localized state that is “non-radiative yet non-bandgap.”

### Classification of bound states in the continuum

**Figure 3:**
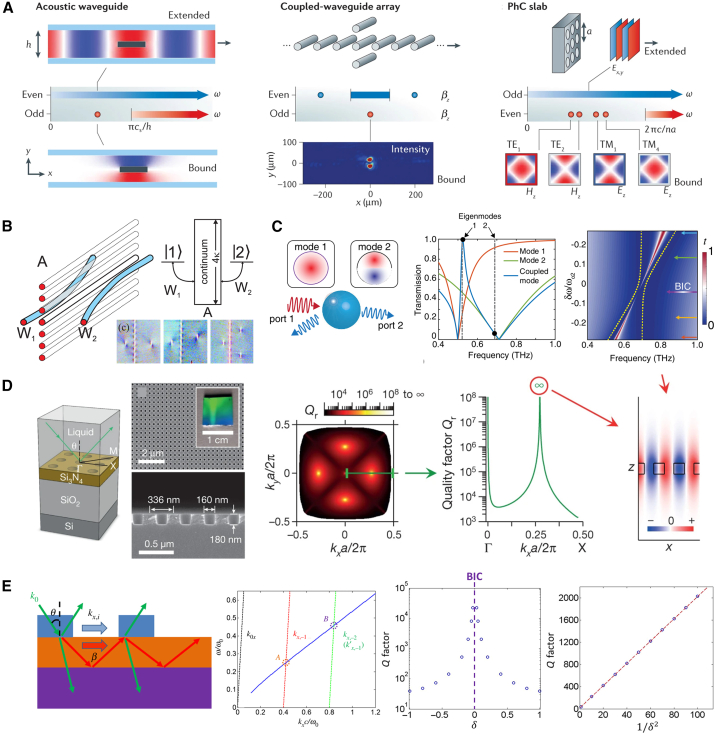
Classification of BICs (A) Symmetry-protected BIC.^21^^,^^32^^,^^44^^,^^50^ Reproduced from ref.^44^ with permission from Springer Nature (2016). (B) Fabry-Pérot BIC. Reproduced from ref.^51^ with permission from Optica Publishing Group (2009). (C) Friedrich-Wintgen BIC. Reproduced from ref.^52^ with permission from Optica Publishing Group (2020). (D) Single-resonance BIC. Reproduced from ref.^53^ with permission from Springer Nature (2013). (E) Momentum-mismatch-driven BIC. Reproduced from ref.^54^ with permission from American Physical Society (2019).

Although BICs lie within the continuous spectrum, they can achieve complete localization and decouple from far-field radiation. Based on the differences in their decoupling mechanisms, BICs can be categorized into two main types: symmetry-protected BICs and accidental (or parameter-tuned) BICs, as shown in Figure 3.

**Figure 4:**
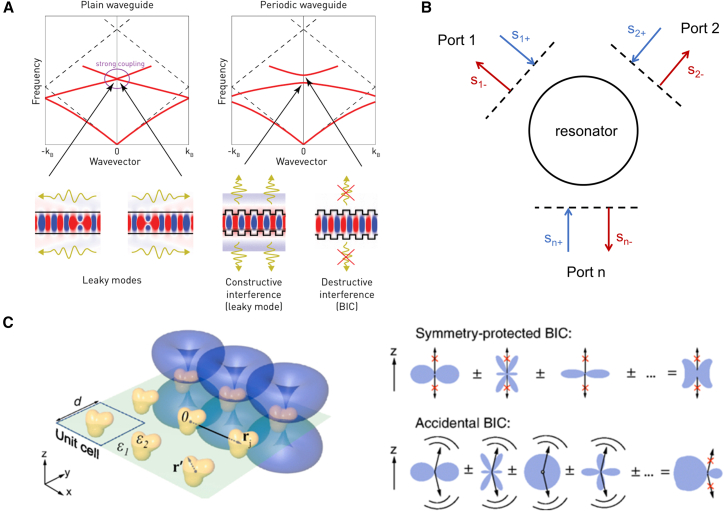
Different BIC analysis theories (A) Band theory analysis of periodic photonic structures. Reproduced with permission from Yuri et al.^46^ (B) Temporal coupled-mode theory.^47^^,^^48^ (C) Multipole moment superposition theory. Reproduced from ref.^49^ with permission from American Physical Society (2019).

Symmetry-protected BICs are determined by the symmetry of the system structure: their radiative modes are orthogonal to eigenmodes with specific symmetries, which prevents coupling between the two. When the structure has certain symmetries (such as C_2_ spatial inversion, mirror symmetry, etc.), even-symmetric resonant modes are orthogonal to odd-symmetric radiative modes, thereby blocking radiative channels. For example, in 2012, Lee et al. found in photonic crystal slabs that for arrays of square cylindrical holes etched in dielectric materials, the structure exhibits periodicity along two mutually perpendicular in-plane directions, and the C_2_ symmetry remains unchanged when rotating around the normal direction. As a result, even-symmetric and odd-symmetric modes related to C_2_ symmetry in the structure are decoupled.^32^ Without considering diffraction, the only radiative wave in the system is a plane wave propagating along the Z-direction, and this plane wave acts as an odd function in the in-plane. Therefore, for any normally incident plane wave, all even eigenmodes of the device are non-radiative, i.e., BICs. As the incident angle shifts to oblique incidence, the plane wave no longer behaves as a strict odd function with respect to C_2_ symmetry. At this point, all even modes lose the protection of C_2_ symmetry and start to radiate.^32^ In 2023, Zhang et al. realized symmetry-protected dual-band BICs in a germanium-based dual-asymmetric periodic grating structure, breaking the limitation of traditional single-band BICs, and significantly expanding the application space of BICs in multi-band optical manipulation scenarios. The two BICs supported by this structure correspond to different operating bands. Flexible adjustment of the bands and *Q*-factors can be achieved by independently regulating the grating and air-gap asymmetry parameters.^45^ Figure 4A further visualizes the generation mechanism of symmetry-protected BICs through three types of systems. In acoustic waveguides, the mirror symmetry of the central plate is consistent with that of the waveguide. Although the localized odd modes below the cut-off frequency overlap with the even-mode continuous spectrum in frequency, they cannot radiate energy due to symmetry-induced decoupling, thus forming BICs.^50^ In coupled optical waveguide arrays, symmetry-protected BICs will couple with radiative channels and transform into leaky resonances when the mirror symmetry is broken by a temperature gradient.^21^ At the Γ-point of photonic crystal slabs, the even-symmetric modes are decoupled from the z-direction radiative plane waves with odd symmetry. Even when located within the continuous spectrum, these even-symmetric modes do not radiate and thus form BICs. When the wave vector deviates from the Γ-point, these modes convert into leaky resonances.^32^ These three cases collectively and intuitively demonstrate the applicability of this mechanism in different wave and material systems.^44^

The core formation mechanism of accidental or parameter-tuned BICs lies in the modulation of parameters related to each radiative channel within the structure. During the modulation process, the energy of each radiative channel undergoes destructive interference in the far-field region, ultimately enabling the entire structure to exhibit a bound state where energy cannot radiate outward. Based on the different compositional forms of resonant modes in the radiative channels, accidental or parameter-tuned BICs can be specifically divided into three categories: Fabry-Pérot BICs, which are formed by the destructive interference between two modes of resonators at different positions; Friedrich-Wintgen BICs, which arise from the destructive interference between two modes of a single resonator at the same position; and single-resonance BICs, which are generated by the independent destructive interference of a single resonant mode.

In 2007, Dreisow et al. experimentally verified the BIC behavior induced by Fabry-Pérot interference in coupled waveguides. In the experiment, the laterally bent waveguides and the waveguide array formed a “dual-reflection/coupling interface” similar to a Fabry-Pérot cavity. The coupling coefficients *κ*_1_(*z*) and *κ*_2_(*z*) of the lateral waveguides varied with the bending profile (e.g., parameters α and dmin), which is analogous to the regulation of cavity length or the spacing between reflective surfaces in an Fabry-Pérot cavity. This variation caused the radiation from different channels to undergo destructive-like interference in the far field, forming a photonic dark state where energy cannot radiate into the continuous state.^51^

In addition, this type of BIC can be formed through a destructive interference mechanism between multiple modes or multiple paths—for instance, the destructive interference of two coupled modes. When the frequencies of the two modes are close and the coupling phases of their radiative channels are opposite, radiative cancellation can be achieved. Chen et al. designed metallic metasurfaces and complementary metasurfaces to realize Friedrich-Wintgen BICs in the terahertz frequency band. Based on the temporal coupled-mode theory, by adjusting the gap of the metasurface unit cells, the inductance-capacitance mode and dipole mode of a single unit in the metallic metasurface, as well as the dual magnetic modes of a single unit in the complementary metasurface, were tuned to have close frequencies, satisfying the Friedrich-Wintgen condition. Destructive interference between the two modes then formed a BIC, without the need to break the C_2_ or mirror symmetry of the structure.^52^

The previous two types of BICs involve two or more coupled resonances, whose radiation cancels each other out to generate BICs. In contrast, single-resonance BICs result from the destructive interference between multiple sets of plane waves within a single Bloch mode. This type of BIC occurs in a single Bloch mode because the multi-order Fourier components of the mode undergo destructive interference in the open channels, leading to suppressed radiation. In 2013, Hsu et al. proposed this type of mode in photonic crystal slabs. Its *Q*-factor exhibits a typical behavior of “maximum-minimum-maximum” with changes in the incident angle, indicating that it is a BIC rather than an ordinary resonant state.^53^

In addition to the classical BICs outlined previously, a distinct class has recently been identified whose formation is driven by momentum mismatch rather than by strict structural symmetry or multi-mode interference.^54^^,^^55^ In these states, light is strongly localized because the dispersion relation is engineered so that the momentum-conservation condition between the localized resonance and the radiation continuum is violated, thereby blocking radiative channels. Momentum-mismatch-driven BICs offer two key advantages: their existence conditions tolerate fabrication errors, conferring superior structural robustness, and the dispersion can be tuned dynamically, enabling reversible switching between BIC and q-BIC states. This mechanism provides a new route for designing broadly tunable optical devices and has already shown promise in optical modulators and high-sensitivity sensors, further expanding the taxonomy and application space of BICs.

At present, research on BICs has gradually evolved from pure theoretical exploration into a frontier interdisciplinary direction. Various studies not only focus on revealing the intrinsic physical mechanisms of BICs but also attempt to control and utilize BICs through platforms such as high-refractive-index media, artificial microstructures, photonic crystals, and topological boundaries.

### Representative milestones motivating modern BIC frameworks

Rather than providing a comprehensive historical survey, this subsection highlights a few representative milestones that have shaped the modern understanding of BICs and motivated the theoretical frameworks and application paradigms discussed in the subsequent sections. The emphasis here is not on chronological completeness, but on identifying conceptual turning points that underpin current BIC-based photonics.

Early theoretical works established the existence of non-radiating states embedded in the radiation continuum through interference and symmetry considerations, laying the foundation for later photonic realizations. These ideas were subsequently translated into optical platforms, most notably photonic crystal slabs and waveguide systems, where symmetry-protected and interference-induced BICs were experimentally observed. Such demonstrations provided the first clear evidence that radiation can be fundamentally suppressed in open optical systems, beyond simple parameter optimization of leaky resonances.^19^^,^^20^^,^^21^^,^^32^^,^^33^^,^^34^^,^^35^^,^^36^^,^^37^ A second milestone was the development of systematic theoretical tools capable of connecting BIC physics with experimentally accessible observables. Temporal coupled-mode theory and scattering-matrix approaches clarified how ideal BICs emerge as singular points in parameter space and how controlled symmetry breaking converts them into quasi-BICs with finite yet ultra-high *Q* factors.^38^^,^^39^^,^^41^^,^^47^^,^^48^^,^^56^^,^^57^^,^^58^^,^^59^^,^^60^^,^^61^^,^^62^^,^^63^^,^^64^ These models established a direct link between structural perturbations, radiative coupling, and resonance linewidth, forming the basis for rational device design rather than empirical tuning, as further elaborated in Section 3. More recently, the recognition of the topological nature of many photonic BICs marked a conceptual shift in the field. The identification of polarization vortices and quantized topological charges in momentum space revealed that BICs are not merely isolated high-*Q* states, but topologically constrained features whose existence and evolution are governed by global invariants.^65^^,^^66^^,^^67^^,^^68^^,^^69^ This insight explains the robustness of BICs under continuous perturbations and enables deterministic control strategies based on topological charge conservation, which play a central role in non-local wavefront modulation and polarization singularity engineering discussed in Section 4.^70^^,^^71^^,^^72^^,^^73^^,^^74^^,^^75^^,^^76^ Together, these milestones delineate the transition of BIC research from isolated theoretical predictions to a unified framework integrating symmetry, interference, and topology. Rather than reviewing the full historical development, the remainder of this article builds upon these conceptual advances to analyze how modern theoretical models inform device-level functionalities and how BIC-based platforms open new opportunities for scalable and multifunctional photonic systems.

## Fundamental theory of BICs

BICs constitute a crucial concept in quantum mechanics and condensed matter physics. They describe bound states formed by particles under the influence of a specific potential field, even though their energy lies within the continuous spectrum. Unlike traditional discrete bound states, BICs typically emerge under specific conditions and exhibit non-localized characteristics. In quantum mechanics, bound states generally refer to states with energy below the scattering threshold, and their wave functions are localized. However, under certain circumstances, particles can still maintain bound states even when their energy falls within the continuous spectrum. Such states are termed BICs, which usually manifest as wave functions with large amplitudes in specific regions and rapid attenuation when moving away from these regions. Typical BICs appear in defect states of quantum waveguides, surface plasmons, topologically protected states, and optical bound states.

The formation of BICs typically relies on specific physical mechanisms, including but not limited to the following two key types. The first is destructive interference. In certain systems, destructive interference occurs between different scattering channels, and this interference suppresses the propagation of modes at specific energy values. Such suppression induces the localization of wave functions, ultimately leading to the formation of bound states. The second is special boundary conditions. For instance, in open systems, particular boundary conditions can inhibit specific modes from coupling with the continuous spectrum. This decoupling effect brought by boundary conditions enables the stable existence of bound states.

BICs can generally be characterized by the time-independent Schrödinger equation or its equivalent form:

(Equation 1) Hψ=Eψ

where *H* is the Hamiltonian operator of the system, ψ is the wave function, and *E* is the energy eigenvalue. For typical bound states, the wave function satisfies the normalization condition

(Equation 2) $${\int \left|\psi \left(x\right)\right|}^{2}dx<\infty$$

However, in the case of BICs, the wave function may not strictly satisfy the normalization condition. Instead, it exhibits a high degree of localization within specific regions.

Another mathematical description is the transmission matrix method. In one-dimensional quantum systems or optical systems, the formation of bound states can be studied through the transmission matrix method, and how specific modes avoid coupling with the continuum can be observed. The transmission matrix method is commonly used in one-dimensional quantum systems to describe the propagation of wave functions in different potential barrier regions:

(Equation 3) $$\left[\begin{array}{l} A_{\text{out}}\\ B_{\text{out}} \end{array}\right]=M\left[\begin{array}{l} A_{\text{in}}\\ B_{\text{in}} \end{array}\right]$$

Condition for no outgoing wave:

(Equation 4) $$B_{out}=0$$

where M is the transmission matrix, describing the scattering characteristics of the system. $A_{\text{in}}$, $B_{\text{in}}$, $A_{\text{out}}$, and $B_{\text{out}}$ are the coefficients of the incident and outgoing waves, respectively.

The third description is based on the Green’s function method. The Green’s function method can be used to analyze localized resonance modes in the continuous spectrum and explain why certain states do not interact with freely propagating states. The Green’s function G(E) is used to analyze the localized resonance modes of the system:

(Equation 5) $$G\left(E\right)={\left(E-H+i\eta \right)}^{-1}$$

where η→0+ is used to ensure causality in the physical sense. BICs correspond to the poles of the Green’s function. Such states do not couple with the continuous spectrum and therefore do not lead to the appearance of scattering resonance peaks:

(Equation 6) det(E−H)=0

In research, band theory in periodic photonic structures, temporal coupled-mode theory, and multipole moment superposition theory are generally adopted to analyze BICs.

Band theory serves as a crucial tool for analyzing BICs in periodic photonic crystals. In photonic crystals, due to the presence of periodic structures, the propagation characteristics of photons are governed by Bloch’s theorem, forming a photonic band structure analogous to electronic energy bands. Band theory derives the relationship between photon frequency and wave vector (i.e., the band structure) by solving the eigenvalue problem of Maxwell’s equations under periodic boundary conditions. Within the band structure, certain specific combinations of frequency and wave vector may correspond to BIC modes. Owing to symmetry or other physical mechanisms, these modes cannot couple to the continuum, thereby forming bound states. Through detailed analysis of the band structure, the existence position, frequency range of BICs, and their interrelationships with other modes can be determined, providing an important theoretical foundation for understanding and designing BICs.

Temporal coupled-mode theory is a dynamic analysis method used to describe the energy coupling and interaction between modes in photonic crystals. When two leaky modes couple in momentum space, their energies exchange and transfer with each other. Temporal coupled-mode theory establishes differential equations to describe such energy coupling and analyzes the constructive and destructive interference effects between modes. In the case of constructive interference, the energies of the two leaky modes mutually enhance, forming a leaky mode with stronger loss. In the case of destructive interference, the energies of the two leaky modes cancel each other out, causing the loss of one mode to disappear and forming a BIC mode. This theoretical method can elaborate the dynamic behavior and energy flow during the formation of BICs, which is helpful for deeply understanding the physical mechanism of BICs and optimizing their design.

The multipole moment superposition theory focuses on analyzing the influence of the excitation and superposition of different multipole moments in metasurfaces on the formation of BICs. In metasurface structures, each unit can support multiple multipole moment modes, such as electric dipoles, magnetic dipoles, and electric quadrupoles. By precisely controlling the geometric parameters and material properties of the metasurface, the excitation intensities and phase relationships of these multipole moments can be adjusted. Spatial superposition of these moments leads to interacting electromagnetic field distributions and complex interference. Under specific conditions, this superposition of multipole moments can cause the outwardly radiated electromagnetic waves to cancel each other out, forming a non-radiative mode, namely a BIC. By analyzing the contributions and interactions of different multipole moments, the multipole moment superposition theory reveals the intrinsic physical mechanism of BIC formation and provides theoretical guidance for the design of metasurfaces with specific optical properties.

### Band theory of periodic photonic structures

Periodic photonic structures are a type of structure with a periodic refractive index distribution in space, and their essence is similar to the lattice structure in solid-state physics. When photons propagate in them, they undergo Bragg scattering similar to that experienced by electrons moving in a solid lattice, thereby forming a band structure, namely photonic bands.^9^^,^^46^^,^^77^ The photonic band theory originates from wave optics and the band theory in solid-state physics. Bragg scattering holds that when light waves propagate in a periodic medium, reflection and interference at the interfaces of different media cause light of specific frequencies to undergo Bragg reflection. The intensity of such Bragg reflection is very strong in certain directions and frequencies, thereby restricting the propagation of light waves and forming photonic band gaps. Due to the periodicity of the structure, according to Bloch’s theorem, the electromagnetic wave solution can be expressed as:

(Equation 7) $$E\left(r\right)=e^{ik\cdot r}u_{k}\left(r\right)$$

where k is the wave vector, and $u_{k}\left(r\right)$ is a function with lattice periodicity, that is:

(Equation 8) $$u_{k}\left(r+R\right)=u_{k}\left(r\right)$$

When Bragg scattering is strong enough in certain directions, light waves within a specific frequency range cannot propagate, that is, a photonic band gap is formed, similar to the electronic band gap in semiconductors. Combining the medium relations:

(Equation 9) D=ε(r)E,B=μ(r)H

In the case of non-magnetic materials μ=1, the electric field E satisfies the eigenvalue equation:

(Equation 10) $$\nabla \times \left(\frac{1}{\varepsilon \left(r\right)}\nabla \times H\right)={\left(\frac{\omega }{c}\right)}^{2}H$$

where *ε* (***r***) is the periodic dielectric constant, i.e., *ε* (***r*** +***R***) = *ε* (***r***), *ω* is the angular frequency of light, and *c* is the speed of light. This equation is analogous to the Schrödinger equation of solid-state physics and can be solved to obtain the photon dispersion relation, namely the photonic band structure.

### Temporal coupled-mode theory

Temporal coupled-mode theory (TCMT) is a mathematical tool used to describe the evolution of coupled oscillators or resonant states in the time domain. It is commonly employed to study mode coupling phenomena in complex optical systems, such as optical fibers, photonic crystals, and resonant cavities. The core idea of TCMT is to treat the system’s resonant modes as a set of coupled oscillators and to study their mutual interactions.^47^^,^^78^^,^^79^^,^^80^ In optical systems, these modes are usually caused by the electromagnetic field distributions in resonant cavities, photonic crystals, or other microstructures. For a system containing multiple modes *ϕ*_i_, *ϕ*_*j*_ whose evolution is driven by external excitation or mutual coupling, TCMT aims to derive the corresponding time-evolution equations. Within TCMT, the total electromagnetic field is expressed as a superposition of multiple modes:

(Equation 11) $$E\left(t\right)=\sum_{i}a_{i}\left(t\right)\phi_{i}$$

where *a*_*i*_(*t*) represents the time-dependent amplitude of the i-th mode, and *ϕ*_*i*_ is the spatial distribution of this mode. It can be assumed that these modes evolve independently without coupling, that is, each mode has an independent resonant frequency *ω*_*i*_, and its time-evolution equation is:

(Equation 12) $$\frac{d^{2}a_{i}\left(t\right)}{dt^{2}}+2\gamma_{i}\frac{da_{i}\left(t\right)}{dt}+\omega_{i}^{2}a_{i}\left(t\right)=0$$

Among them, *γ*_*i*_ is the damping factor of the mode, and *ω* is the resonant frequency of the mode.

When multiple modes are coupled, their evolution is no longer independent. The coupling term *β*_*ij*_ describes the coupling strength between mode *i* and mode *j*. The introduction of coupling makes the time-evolution equation of each mode become:

(Equation 13) $$\frac{d^{2}a_{i}\left(t\right)}{dt^{2}}+2\gamma_{i}\frac{da_{i}\left(t\right)}{dt}+\omega_{i}^{2}a_{i}\left(t\right)=-\sum_{j\neq i}\beta_{ij}a_{j}\left(t\right)$$

Consequently, each mode is influenced by both its own dynamics and those of all other modes.

To solve the system, it is assumed that during coupling the amplitude *a*_*i*_(*t*) of each mode can be written as a superposition of sinusoidal functions whose frequencies are close to the respective mode resonances. The general solution of the system is then assumed to be:

(Equation 14) $$a_{i}\left(t\right)=A_{i}e^{i\omega_{i}t}+B_{i}e^{-i\omega_{i}t}$$

where *A*_*i*_ and *B*_*i*_ are constants. Substituting the above ansatz into the time-evolution equation for each mode yields a set of coupled equations. The solutions of these equations can describe the energy exchange and evolution process between each mode in the system. For practical simplicity, the coupling strength *β*_*ij*_ is assumed to be constant and the coupling between modes is linear. The coupled equations then take the form:

(Equation 15) $$\frac{d^{2}a_{i}\left(t\right)}{dt^{2}}+2\gamma_{i}\frac{da_{i}\left(t\right)}{dt}+\omega_{i}^{2}a_{i}\left(t\right)=-\sum_{j\neq i}\beta_{ij}a_{j}\left(t\right)$$

The solutions of this set of coupled equations can be obtained numerically or analytically, with the choice depending on the specific characteristics of the system.

Assume that the optical system supports two interacting modes (as in photonic crystals or microcavities), denoted Mode 1 and Mode 2. The system Hamiltonian *H* is then expressed as:

(Equation 16) H=Ω+Γ

where Ω is the time-domain frequency matrix of coupled modes, and Γ is the loss matrix representing mode coupling. It is specifically expressed as follows:

(Equation 17) $$H=\left[\begin{array}{cc} \omega_{1} & \alpha \\ \alpha & \omega_{2} \end{array}\right]+\left[\begin{array}{cc} \gamma_{1} & \kappa \\ \kappa & \gamma_{2} \end{array}\right]$$

The coupling and loss terms of the system are encoded in the matrices Γ and Ω. The elements in the matrices represent the eigenfrequencies and coupling strengths of different modes. Here, *ω*_1_ and *ω*_2_ are the frequencies of the two modes, respectively, α is the coupling coefficient between the modes, *γ*_1_ and *γ*_2_ are the damping factors of the modes respectively, and *κ* is the coupling constant between the modes.

To obtain a BIC, the eigenvalue problem is first solved to identify the conditions under which the eigenvalues correspond to bound rather than free states. Substituting the previous Hamiltonian into the characteristic equation yields:

(Equation 18) |H−ωI|=0

That is:

(Equation 19) $$\left|\begin{array}{cc} \omega_{1}-\omega & \alpha \\ \alpha & \omega_{2}-\omega \end{array}\right|-\left|\begin{array}{cc} \gamma_{1} & \kappa \\ \kappa & \gamma_{2} \end{array}\right|=0$$

In the case of BIC formation, some solutions of the system become real numbers. Therefore, it can be decomposed into two solutions *ξ*_1_ and *ξ*_2_, which are in the form of:

(Equation 20) $$\xi_{1}=A-i\gamma_{1}-i\gamma_{2}$$

(Equation 21) $$\xi_{2}=B$$

At this time, algebraic equations can be used to obtain the relationship between A and B, as follows:

(Equation 22) $$A+B=\omega_{1}+\omega_{2}$$

(Equation 23) $$AB=\omega_{1}\omega_{2}-\alpha^{2}$$

By substituting the above equations, the relational expression between A and B can be obtained:

(Equation 24) $$B=\frac{\omega_{1}\gamma_{2}+\omega_{2}\gamma_{1}-2p\alpha \sqrt{\gamma_{1}\gamma_{2}}}{\gamma_{1}+\gamma_{2}}$$

Next, using the previous relational expression to solve for the eigenfrequency *ω*:

(Equation 25) $$\omega =\frac{1}{2}\left[\omega_{1}+\omega_{2}\pm \sqrt{{\left(\omega_{1}-\omega_{2}\right)}^{2}+4\alpha^{2}}\right]$$

To satisfy the BIC condition, B needs to be a constant. This means the following relationship must be satisfied:

(Equation 26) $$\sqrt{{\left(\omega_{1}-\omega_{2}\right)}^{2}+4\alpha^{2}}=\pm \left[\left(\omega_{1}-\omega_{2}\right)\left(\gamma_{1}-\gamma_{2}\right)+4p\alpha \sqrt{\gamma_{1}+\gamma_{2}}\right]$$

At this point, the BIC condition for the system is written as:

(Equation 27) $$p\alpha \left(\gamma_{1}-\gamma_{2}\right)=\sqrt{\gamma_{1}\gamma_{2}}\left(\omega_{1}-\omega_{2}\right)$$

This implies that the emergence of BIC depends on the relationship between the mode coupling strength, damping factors, and frequency differences in the system. Through the temporal coupled-mode theory and the above mathematical derivation, the formation conditions of BIC can be obtained. Based on these conditions, optical systems that satisfy the BIC conditions can be designed. For example, tuning the mode-coupling strength and damping rates allows controlled BIC emergence, giving rise to bound-state formation or energy localization in photonic devices. The derivations provide a general BIC-analysis framework based on temporal coupled-mode theory and are applicable to the design and optimization of microcavities, photonic crystals, and related photonic systems.

While the theoretical frameworks introduced previously—namely band theory, TCMT, and multipole interference analysis—provide a rigorous understanding of the formation of BICs, their true significance lies in how they translate into concrete device-design strategies. In practical photonic systems, these models establish direct and quantitative links between structural parameters and measurable device performance, thereby enabling rational design rather than empirical trial-and-error optimization. Among these approaches, temporal coupled-mode theory plays a particularly central role in bridging theory and application. By explicitly describing the coupling between resonant modes and radiation channels through external coupling rates and intrinsic loss terms, TCMT offers an intuitive yet quantitative framework for engineering the linewidth, bandwidth, and sensitivity of BIC-enabled devices. For example, in refractive-index sensors based on quasi-BIC resonances, TCMT reveals that the sensing bandwidth and detection limit are governed by the balance between radiative leakage and material absorption. By deliberately introducing a controlled symmetry breaking, the radiative decay rate can be tuned to match intrinsic losses, achieving critical, or near-critical coupling. This condition simultaneously maximizes field enhancement and optimizes spectral linewidth, thereby enhancing both sensitivity and signal-to-noise ratio in practical sensing platforms. A similar design principle applies to nonlinear optical devices. In BIC-enhanced frequency converters, TCMT clarifies how the nonlinear conversion efficiency depends not only on the local field enhancement associated with high-*Q* resonances, but also on the spectral overlap between interacting modes. By tailoring the coupling coefficients and detuning parameters within the TCMT framework, designers can control the effective interaction bandwidth and phase-matching conditions, enabling efficient nonlinear processes under realistic pumping conditions. This perspective explains why quasi-BICs, rather than ideal BICs, are often preferred in experiments: a finite but well-controlled radiative coupling allows efficient energy extraction while retaining strong field confinement.

### Multipole moment superposition theory

In periodic optical structures, each minimum structural unit can support multiple multipole moment modes, including electric dipole, magnetic dipole, and electric quadrupole contributions. The excitation and superposition of these multipole moment modes can be controlled by adjusting the geometric parameters and material properties of the structure. Spatial superposition of these moments leads to interacting electromagnetic field distributions and complex interference.^49^^,^^71^^,^^81^

The electromagnetic multipole moment theoretical model is a multipole decomposition method, which expresses the radiation field as the sum of spherical harmonic vectors based on the symmetry of the lattice and unit structure. Specifically, the far-field radiation electric field can be expressed as:

(Equation 28) $$E\left(r\right)=C\;\exp\left(ik_{1}r\right)\sum_{p_{i},p_{t},m,n}i^{-n}{\tilde{D}}_{p_{i}p_{t}mn}\left[Y_{p_{i}p_{t}mn}\left(\frac{k_{1}}{k_{1}}\right)\right]$$

where *C* is a constant; *p*_*i*_, *p*_*t*_, *m*, and *n* are parameters of the multipole; $\tilde{D}$ is the multipole expansion coefficient; and *Y* is the spherical harmonic function.^49^

In periodic optical structures, the radiation from each unit cell is described by the excitation of multipole moments. For example, the unit-cell radiation field is expressed as a superposition of electric dipole, magnetic dipole, electric quadrupole, and higher-order contributions. When multiple unit cells form a periodic lattice, their multipole moments superimpose spatially. This superposition is described by an expansion in vector spherical harmonics, as given in Equation 28.

Under specific symmetry conditions, multipole-moment superposition can lead to directional radiation cancellation. For example, in symmetry-protected BICs, all multipole parameters at the Γ point are non-zero, yet symmetry forces the z-component of the far-field electric radiation to vanish, forming a BIC. Away from Γ, the superposition of multipole components in the z axis direction is generally non-zero. However, at special *k*_*i*_ points, by adjusting the phase and amplitude of the multipole moments, the sum of each multipole moment component in Equation 28 in the z axis direction can be made zero, thereby forming an accidental BIC.^49^^,^^82^

The multipole moment superposition theory reveals the intrinsic physical mechanism of BIC formation by analyzing the excitation and superposition of different multipole moments in metasurfaces. Through precise control of multipole moments, BICs can be manipulated and exploited in metasurfaces, providing a key theoretical foundation for the design and optimization of micro- and nano-scale optical devices.

### Topological charge and polarization vortices of BICs

A fundamental theoretical aspect complementing symmetry-protection and interference models of BICs (BICs) is their intrinsic topological nature, which manifests as polarization vortices in momentum space. This perspective was established when it was shown that each BIC corresponds to a singularity in the far-field polarization field of a Bloch mode, around which the polarization orientation winds by an integer multiple of 2*π*. This integer winding number is defined as the topological charge of the BIC and provides a geometric, structure-independent criterion for identifying and classifying BICs. Unlike conventional resonant states, which can be modified or eliminated through gradual parameter variations, BICs exhibit topological robustness: their defining polarization singularities cannot be removed unless they encounter another BIC carrying an opposite charge, enabling pairwise annihilation. As a result, BICs can shift continuously within the Brillouin zone when the structure is perturbed—for example, through changes in geometric asymmetry, refractive-index modulation, or substrate loading—but their existence cannot be eliminated through such continuous deformations. Instead, BICs follow deterministic evolutionary trajectories governed by topological constraints, and their total topological charge remains invariant throughout the tuning process.

The polarization vortex associated with a BIC provides an intuitive physical picture: although the radiation amplitude vanishes at the BIC point, the far-field polarization remains well defined and exhibits a characteristic rotational texture in reciprocal space. Mapping the far-field polarization orientation or Stokes parameters of the Bloch modes enables direct visualization of these vortices, offering an experimentally accessible means of identifying BICs and verifying their topological characteristics. The vortex orientation distinguishes between positive and negative charges, while configurations involving higher-order or merged vortices arise when multiple singularities coalesce under structural tuning. These topological features endow BICs with remarkable resilience against fabrication imperfections, since small structural deviations merely perturb the position of the vortex without destroying its topological identity.

The conservation of topological charge also provides a unified explanation for a range of phenomena observed in BIC-based photonic systems. Under structural modifications, isolated BICs can migrate through the Brillouin zone, split into multiple lower-order singularities, or merge to form higher-order BICs, yet the total winding number remains conserved. This behavior underlies the robustness of high-*Q* resonances in photonic crystals and metasurfaces and offers a predictive framework for designing tunable BIC platforms. Furthermore, the topological configuration of BICs directly influences device-level properties. In BIC lasers, the far-field vortex structure governs the polarization state and orbital angular momentum of emitted beams. In chiral and spin-orbit photonics, the sign and magnitude of the topological charge determine the strength and direction of circular-dichroism responses. In nonlinear optics, the interplay between vortex topology and field localization modulates nonlinear conversion efficiency. In beam-shift phenomena and photonic spin Hall effects, the geometric phase accumulated around the vortex plays a decisive role in determining the magnitude of the displacement. Thus, the topological framework not only deepens the fundamental understanding of BICs but also enriches their functional landscape, providing new degrees of freedom for engineering high-performance and reconfigurable photonic devices.

## Cutting-edge applications of BICs in periodic optical systems

In terms of applications, q-BICs have become a research focus due to their high *Q*-factors and controllable radiation characteristics. By designing systems near the ideal BIC conditions, q-BIC modes with significantly higher *Q*-factors than ordinary resonant modes can be achieved. Coupled-mode theory offers an effective modeling framework for q-BICs: resonant modes are coupled to radiation channels, and the introduction of complex frequencies and coupling coefficients yields a quantitative relation between the *Q*-factor and structural parameters. This provides the theoretical foundation for designing high-performance photonic and acoustic devices.

BICs have attracted intense attention not only as a fundamental concept in theoretical optics but also for their exceptional optical performance, which enables novel opportunities for a wide range of practical applications. In various optical devices, BICs enable resonances with extremely high *Q*-factors and strong local electric-field enhancement, providing a solid foundation for designing efficient, low-loss, and compact photonic components. In recent years, researchers have extended BIC mechanisms to filters,^83^^,^^84^^,^^85^ lasers,^86^^,^^87^^,^^88^^,^^89^^,^^90^^,^^91^^,^^92^^,^^93^^,^^94^^,^^95^^,^^96^ nonlinear optics,^64^^,^^97^^,^^98^^,^^99^^,^^100^^,^^101^^,^^102^^,^^103^^,^^104^^,^^105^ sensors,^106^^,^^107^^,^^108^^,^^109^^,^^110^^,^^111^^,^^112^^,^^113^ terahertz devices,^114^^,^^115^^,^^116^^,^^117^^,^^118^^,^^119^ edge detection,^120^^,^^121^^,^^122^^,^^123^^,^^124^ and many other fields, continuously expanding their functional boundaries and practical application potential.

### Research progress of high-performance miniaturized BIC lasers

**Figure 5:**
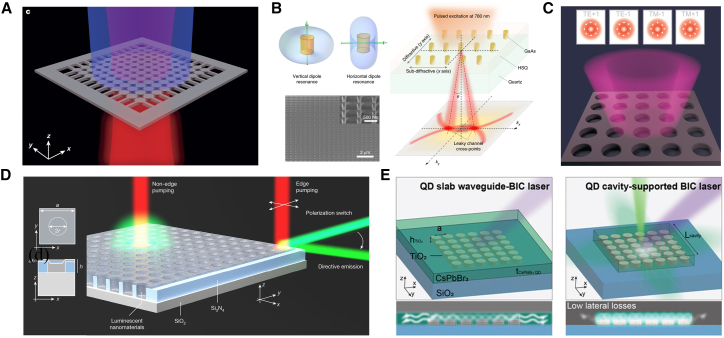
Applications of BIC in laser design (A) BIC-based laser emission realized in photonic crystals. Reproduced from ref.^86^ with permission from Springer Nature (2017). (B) Directional laser output based on semiconductor nanoantenna arrays. Reproduced from ref.^87^ with permission from Springer Nature (2018). (C) Laser with tunable polarization state and modal distribution. Reproduced from ref.^88^ with permission from John Wiley and Sons (2022). (D) Supercritical BIC-driven upconversion laser. Reproduced from ref.^89^ with permission from Springer Nature (2024). (E) Perovskite quantum dot laser. Reproduced from ref.^91^ with permission from John Wiley and Sons (2024).

In the field of lasers, researchers exploit the high-*Q* factor and strong field localization of BICs to enhance laser performance and overcome traditional limitations. Applications of BICs in laser design are illustrated in Figure 5. Symmetry-protected BIC lasers rely on strict structural symmetry to block radiation channels, and their *Q*-factors are directly determined by the integrity of the symmetry. At high-symmetry points, even-symmetric modes are orthogonal to odd-symmetric radiating plane waves, enabling theoretically infinite *Q*-factors. In 2017, Kodigala et al. first realized laser emission based on symmetry-protected BICs in photonic crystals, greatly reducing the lasing threshold and verifying the effectiveness of BICs in enhancing optical feedback.^86^ In 2018, Ha et al. converted the symmetry-protected BIC originally at the Γ point into a high-*Q* leaky resonance by breaking structural symmetry, achieving directionally controllable quasi-BIC lasing.^87^ In 2022, Mohamed et al. demonstrated four types of BIC lasing with controllable topological charges in a C_4_ᵥ-symmetric photonic crystal slab. Through far-field polarized vortex imaging and multipole decomposition, the topological properties of BICs were revealed, and the polarization manipulation capability of multimode BIC lasers was demonstrated, expanding the design space for topological lasers.^88^ In 2025, the Wang group introduced air holes through tetrameric nanodisks to break in-plane symmetry, exciting dual-mode q-BICs in the telecommunication band. Their BIC characteristics originate from symmetry-protected dark states, and polarization-independent dual-wavelength lasing was achieved by adjusting asymmetric parameters, laying the foundation for multifunctional metasurface lasers.^95^

However, symmetry requirements also introduce significant limitations: the frequency of their dark states is constrained by symmetry, resulting in a limited tuning range. Once structural symmetry is broken, the *Q*-factor of BIC modes decreases significantly. This imposes extremely high demands on fabrication precision, making large-scale production, and flexible wavelength manipulation challenging.

Accidental BIC lasers achieve radiation suppression through destructive interference of multiple modes, with their *Q*-factors continuously tunable via structural parameters and offering higher design flexibility. In 2024, the Schiattarella group constructed an accidental quasi-BIC laser based on the Friedrich-Wintgen mechanism, achieving eight-order upconversion luminescence enhancement through super critical coupling and demonstrating efficient coupling between BICs and multiphoton processes for the first time.^89^ In the same year, Xing et al. fabricated isolated cavity structures using CsPbBr_3_ quantum dots and developed accidental quasi-BIC lasers via a solution method, achieving ultra-small single-mode lasing by suppressing lateral leakage through boundary engineering.^91^ In 2025, Gu et al. demonstrated a silicon-integrated perovskite accidental BIC laser by fabricating FAPbI_3_ microdisks with ultra-smooth sidewalls via focused ion beam (FIB) technology. Utilizing the BIC mechanism to suppress optical leakage between the perovskite and silicon substrate, a high *Q*-factor of 4850 was achieved at 822 nm.^94^

More importantly, the merged BIC strategy has become a key route to overcoming the performance bottlenecks of accidental BICs. By engineering multiple isolated accidental BICs in momentum space to coincide within the same frequency band, hybrid states are created through modal superposition, simultaneously boosting both the *Q*-factor and robustness. For instance, in 2025, the Cui group incorporated hexagonally rotationally symmetric, daisy-shaped holes into terahertz quantum-cascade lasers, forcing multiple modes into accidental degeneracy. The cooperative action of the flat band and multi-BICs suppresses three-dimensional leakage, yielding stable single-mode lasing with a high *Q*-factor and ultra-low threshold in an ultra-compact cavity.

Accidental BIC lasers offer high design flexibility and continuously tunable *Q*-factors via structural parameters, making them suitable for diverse applications. Furthermore, they provide broad wavelength tuning, achievable over a wide range by adjusting structural parameters. However, they still demand high fabrication precision. Additionally, the multimodal nature of accidental BIC lasers may induce mode competition, compromising the stability of single-mode operation.

Despite the significant progress made by BIC lasers in directionality, low threshold, and integration, they still face challenges such as limited dynamic control capabilities, environmental sensitivity, and insufficient thermal stability. Future work should enhance dynamic performance through electrical, thermal, or strain tuning, and should explore the design and integration of large-scale BIC laser arrays. BIC lasers are expected to play a core role in on-chip light sources, tunable lasers, and nonlinear laser devices.

### Nonlinear optical enhancement and applications based on BIC

**Figure 6:**
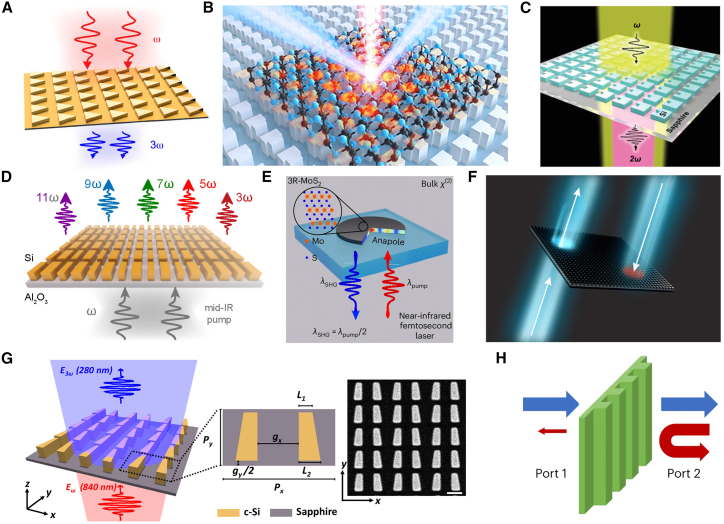
Applications of BIC in nonlinear optics (A) BIC-enhanced third-harmonic generation. Reproduced from ref.^64^ with permission from American Chemical Society (2019). (B) BIC enabling efficient second-harmonic generation (SHG) output. Reproduced from ref.^98^ with permission from American Chemical Society (2021). (C) Coupling of silicon slotted nanostructures with BIC states enhances nonlinear processes. Reproduced from ref.^99^ with permission from John Wiley and Sons (2022). (D) BIC-enhanced metasurface generating higher-order harmonics. Reproduced from ref.^100^ with permission from American Chemical Society (2022). (E) Transition metal dichalcogenide nanoplates enabling efficient third-harmonic generation. Reproduced from ref.^102^ with permission from Springer Nature (2024). (F) Nonlinear metasurface-enabled nanoscale optical nonreciprocal devices. Reproduced from ref.^125^ with permission from Springer Nature (2024). (G) BIC resonance enhanced frequency conversion in the deep ultraviolet band. Reproduced from ref.^103^ with permission from American Chemical Society (2024). (H) Passive nonreciprocal modulation based on thermal nonlinearity. Reproduced from ref.^104^ with permission from Springer Nature (2023).

In the field of nonlinear optics, the high *Q*-factor and strong local field of BIC provide a new approach for improving nonlinear efficiency, as shown in Figure 6. In 2019, Koshelev et al. excited q-BIC through symmetry breaking, significantly enhancing nonlinear processes such as frequency doubling and mixing, which highlighted the field enhancement advantage of BIC.^64^ The Liu team coupled two-dimensional highly nonlinear materials such as GaSe with the BIC platform in 2019 and 2021, and realized efficient second-harmonic generation (SHG) under continuous-wave pumping, providing a new solution for low-power nonlinear devices.^97^^,^^98^ In 2022, Fang et al. used silicon grooved nanostructures to couple with BIC states, overcoming the limitation of low nonlinear efficiency of silicon materials.^99^ Zograf et al. constructed a high-order harmonic generation platform in dielectric metasurfaces and systematically studied the enhancement effect of BIC on high-order nonlinear responses.^100^ The Carletti team significantly improved the third-order nonlinear efficiency based on BIC in AlGaAs nanostructures, demonstrating the potential of III-V semiconductors in nonlinear BIC devices.^101^

Since 2024, relevant research has been further deepened. Zograf et al. reported in Nature Photonics a strongly coupled BIC structure based on nanodisks of two-dimensional materials with high refractive index such as WS_2_, achieving an order-of-magnitude improvement in third-harmonic generation efficiency.^102^ Tripathi et al. employed nonlinear metasurfaces to realise nanoscale optical non-reciprocal devices, demonstrating the potential of BICs for asymmetric optical signal processing.^125^ The Wang group enhanced dipole-quadrupole interference via a symmetry-broken plasmonic dimer, boosting SHG efficiency under non-resonant conditions and expanding the nonlinear application space of q-BICs.^126^ In the same year, Abdelraouf et al. proposed a modal phase-matching mechanism that achieves high-*Q* resonance-enhanced frequency conversion in the deep-ultraviolet region.^103^ The Cotrufo team realised passive non-reciprocal control based on thermal nonlinear effects, offering a new route for developing dynamic nonlinear metasurface devices.^104^

Research on BIC in nonlinear optics shows diversified development from material innovation to mechanism expansion. It not only greatly improves the nonlinear conversion efficiency but also promotes the innovation of integrated, dynamically tunable, and nonreciprocal functional devices. In the future, its application boundaries are expected to expand further through integration with heterogeneous platforms, topological photonics, and quantum light sources.

Research on nonlinear BICs is transitioning from principle verification to system integration and multifunctional implementation, providing a crucial physical foundation for efficient frequency conversion, optical computing, and information processing. Although BICs have markedly enhanced nonlinear frequency conversion, several bottlenecks remain to be addressed. First, most existing BIC structures are statically designed and lack real-time dynamic tunability. Second, high conversion efficiency is often accompanied by insufficient modal selectivity, limiting precise control of the output spectrum. Third, in complex integrated environments, device structural complexity and material stability remain restrictive factors for practical applications.

### Optical chirality and polarization control based on BIC

**Figure 7:**
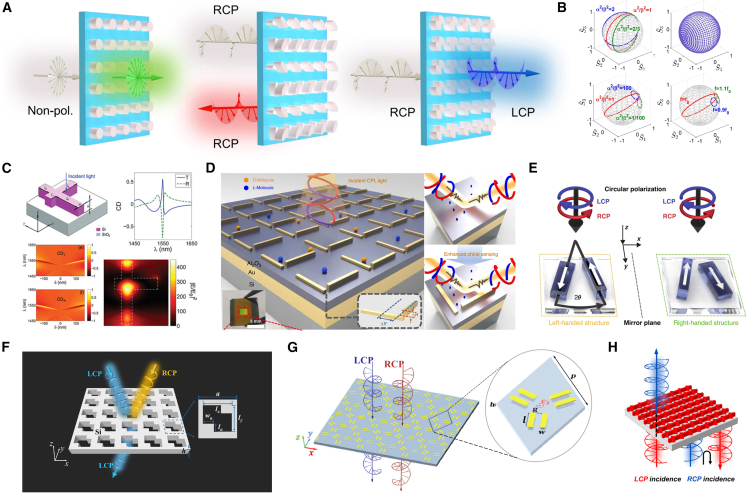
Applications of BIC in polarization control and chiral optics (A) Circular dichroism signal modulation. Reproduced from ref.^129^ with permission from Springer Nature (2022). (B) Omnidirectional polarization control. Reproduced from ref.^130^ with permission from Springer Nature (2022). (C) BIC-enhanced chiral sensitivity. Reproduced from ref.^131^ with permission from John Wiley and Sons (2021). (D) Circular dichroism chiral metasurface molecular recognition. Reproduced from ref.^132^ with permission from Springer Nature (2023). (E) Maximum optical chirality enhancement by BIC. Reproduced from ref.^133^ with permission from Springer Nature (2023). (F) Multiple BIC enhanced structural circular dichroism response. Reproduced from ref.^134^ with permission from Chinese Laser Press (2023). (G) Dual BIC achieving maximum intrinsic chiral response. Reproduced from ref.^136^ with permission from Chinese Laser Press (2024). (H) Broadband bidirectional modulation of topological chiral properties. Reproduced from ref.^137^ with permission from American Chemical Society (2024).

In polarization and chiral optics, BIC structures—owing to their tunable symmetry and field-localization capability—have become an ideal platform for studying chiral enhancement, polarization conversion, and circular-dichroism control, as shown in Figure 7. In 2020, Kivshar’s group introduced the concept of “quasi-BICs with maximum chirality” and fabricated a dielectric-bar-dimer metasurface. By breaking both in-plane and out-of-plane symmetry and tuning the geometric parameters, the structure transmits one circular polarization almost completely while resonantly absorbing the other, producing a unity-amplitude peak in the circular-dichroism spectrum. This demonstration established a foundation for chiral BIC optical devices.^127^ In 2021, Overvig et al. theoretically elucidated the generation mechanism of chiral q-BIC and proposed general design criteria, thereby systematizing chiral-photonics theory.^128^ The following year, the Shi team demonstrated the first tunable chiral BIC structure based on symmetry breaking, achieving a strong circular-dichroism response and verifying the link between structural and optical chirality.^129^ In the same year, the Alù team proposed an omnidirectional polarization-control scheme that achieves efficient, lossless conversion between linear and circular polarizations, highlighting BIC’s potential for advanced polarization control.^130^ In chiral sensing, Kim et al. first experimentally demonstrated BIC-enhanced chiral sensing in 2021. By breaking in-plane symmetry, they significantly improved polarization selectivity and detection sensitivity.^131^ In 2023, the Xu team combined BIC resonance with vibrational circular dichroism (VCD) to develop a high-sensitivity chiral metamaterial for molecular “fingerprint” recognition, increasing detection sensitivity several-fold and expanding BIC applications in biological sensing and spectral analysis.^132^

To further enhance chiral performance, in 2023, the Yuri Kivshar group overcame the limitation of in-plane structures by introducing the z axis degree of freedom, achieving a three-dimensional BIC design that combines strong chiral response with directional selectivity.^133^ Duan et al. constructed a topology-enhanced chiral platform by stacking multiple BIC states in a photonic-crystal slab, offering a new route to high-precision circular-polarization detection.^134^ In the same year, Xiao Shumin and the Yuri Kivshar team reported in Nature an intrinsic chiral BIC based on inclined-perturbation metasurfaces, marking a paradigm shift in the field.^135^ In 2024, Qi et al. proposed achieving maximum intrinsic chirality via dual-BIC resonance excitation, demonstrating strong circular dichroism in a single-layer metasurface.^136^ The Wang team developed a topological chiral broadband bidirectional control system that is passive, wideband, and easily integrated, advancing the practical deployment of BICs in chiral optical-field control.^137^

Although BIC-driven chiral photonic devices exhibit excellent performance, they still suffer from narrow response bandwidth, sensitivity to temperature and fabrication errors, and insufficient functional integration. Next stage research will focus on the synergistic integration of asymmetric topological structures and higher order BIC modes to create chiral systems with broadband response and controllable characteristics. Furthermore, merging multiple functions including polarization conversion, chiral detection and light modulation on a single chip will propel BIC chiral platforms toward high integration, reconfigurability and on chip system implementation.

### Research on the application of BIC in terahertz wave dynamic control and device integration

**Figure 8:**
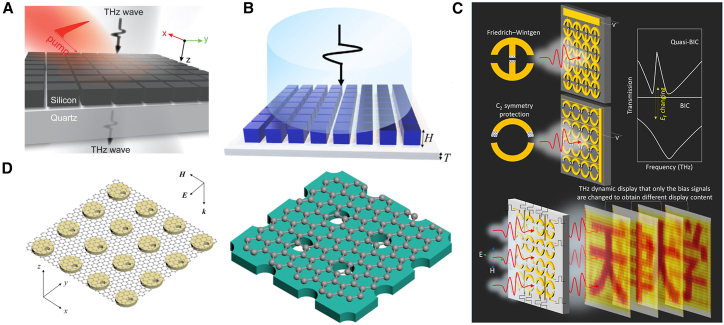
Studies of BIC in the terahertz band (A) All-dielectric reconfigurable terahertz modulator. Reproduced from ref.^114^ with permission from John Wiley and Sons (2019). (B) Resonance switching achieved by BIC. Reproduced from ref.^115^ with permission from Elsevier (2022). (C) BIC switching achieved by graphene-metal hybrid structure. Reproduced from ref.^116^ with permission from Elsevier (2021). (D) Metasurface perfect absorber. Reproduced from ref.^118^^,^^119^ with permission from American Physical Society (2020).

In micro- and nano-antenna applications, the BIC mechanism exhibits strong electromagnetic-control potential from the mid- and far-infrared to the terahertz range, as shown in Figure 8. Han et al. proposed an all-dielectric tunable metasurface structure, which combines active control function with BIC state for the first time, realizing low-loss and reconfigurable terahertz wave modulation. This design realizes the excitation and suppression of BIC modes through dynamic adjustment of symmetry, offering a new route for on-chip integrated terahertz modulators.^114^ The Yue team fabricated a silicon-based metasurface supporting BIC resonance and switched high-*Q* resonance states in the terahertz band by tuning structural parameters.^115^ Li et al. further introduced a graphene-metal hybrid structure and achieved electrically controlled, reversible conversion between BIC and q-BIC states, thereby realizing an intelligent switching terahertz modulator.^116^ Zhang et al. designed a terahertz planar chiral metasurface based on accidental BIC. Without breaking the key symmetry, the metasurface realizes the C-point regulation at the Γ-point and the full-range tunability of the CD value, and simultaneously possesses high *Q*-factor and structural robustness.^117^ These studies not only verify the tunability and practicability of BIC in the terahertz frequency band, but also show that on two-dimensional material platforms such as graphene, BIC structures can achieve dynamic response, and have good thermal stability and wide-band operation capability, providing a key technical path for the next-generation terahertz communication, sensing, and imaging systems.

Wang and Xiao et al. designed THz metasurface absorbers with high absorption under the critical coupling condition, respectively. By tuning the magnetic-dipole resonance induced by the BIC, they enhanced the strength of light-matter interaction and expanded the tuning space of THz functional devices. The absorbers exhibit strong directional and polarization selectivity, offering a structural platform for THz signal enhancement and detection.^118^^,^^119^ However, terahertz BIC research remains at the stage of single-function verification and demonstration. There is an urgent need to extend BIC-based THz devices to dual- and multi-frequency dynamic response platforms, to realize frequency tunable filtering and modulation, and to promote system level integration and stability testing of THz BIC structures in communications, nondestructive testing, and imaging. As a key bridge between fundamental photonics and engineering applications, the terahertz BIC platform is expected to play a pivotal role in next-generation intelligent sensing, terahertz communications, and high-speed imaging.

### Research progress on BIC-based beam shift enhancement

**Figure 9:**
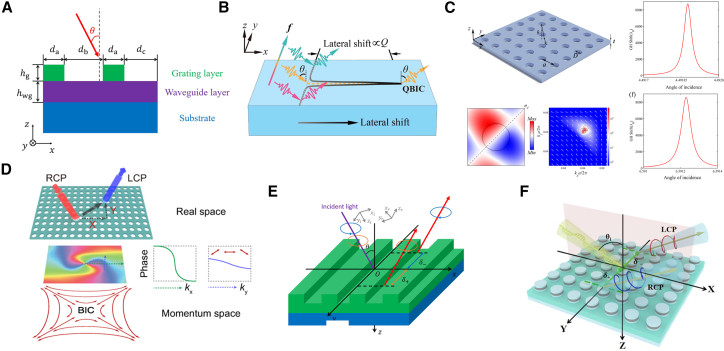
Research on BIC-based beam shift enhancement (A) Dual-periodic gratings for realizing dual quasi-BICs to enhance the GH shift. Reproduced from ref.^139^ with permission from American Physical Society (2021). (B) q-BICs-assisted giant GH shift in the terahertz band. Reproduced from ref.^142^ with permission from Elsevier (2025). (C) Merged BIC metasurfaces for enhancing the GH shift. Reproduced from ref.^140^ with permission from AIP Publishing (2023). (D)The photonic spin Hall effect of light induced by topological vortices of BICs. Reproduced from ref.^144^ with permission from American Physical Society (2022). (E) Polarization-dependent q-BICs in hybrid grating-waveguide structures for enhancing PSHE. Reproduced from ref.^145^ with permission from American Physical Society (2023). (F) High *Q*-factor BIC metasurfaces for PSHE enhancement. Reproduced from ref.^146^ with permission from American Physical Society (2025).

The q-BICs offer a new route for enhancing beam shifts due to their ultra-high *Q*-factors and strong field localization, as shown in Figure 9. Since the first demonstration of quasi-BIC-assisted giant Goos-Hänchen (GH) shift enhancement in 2019,^54^ research has expanded rapidly, achieving GH shift enhancement in multiple scenarios and extending to photonic spin Hall effect (PSHE) enhancement.

The GH shift describes the lateral displacement of a light beam upon total internal reflection. By virtue of the abrupt variation of reflection phase in the resonant region, q-BICs enable the GH shift to break the limitations of traditional mechanisms, achieving giant enhancement on the order of the wavelength while maintaining the advantage of high reflectivity.^54^^,^^138^^,^^139^^,^^140^^,^^141^^,^^142^ In 2019, Wu et al. first realized reflective q-BICs in a four-part periodic grating-waveguide hybrid structure, enhancing the GH shift to four orders of magnitude of the wavelength.^54^ In 2025, q-BIC-assisted giant GH shifts were experimentally observed for the first time in the terahertz band. This achievement not only provides a novel real-space characterization method for q-BICs but also lays a foundation for the development of high-performance devices in the terahertz band, such as sensors and wavelength multiplexers.^142^ In 2021, the same group demonstrated dual q-BICs, simultaneously achieving large positive and negative GH shifts by selectively exciting forward- and backward-propagating guided-mode resonances.^139^ In 2023 Chen et al. introduced the merged BIC mechanism into a two-dimensional photonic crystal slab, enhancing the *Q*-factor by two orders of magnitude and further increasing the GH shift.^140^

The photonic spin Hall effect (PSHE) originates from the spin-orbit coupling of light and manifests as the lateral splitting of left- and right-circularly polarized components. The ultra-high *Q*-factor resonance of q-BICs not only amplifies the phase gradient but also modulates spin-dependent phase variations through polarization-dependent characteristics, enabling an order-of-magnitude enhancement of the lateral displacement in the PSHE.^143^^,^^144^^,^^145^^,^^146^ In 2019, Jiang et al. revealed BIC enhancement in epsilon-near-zero (ENZ) materials, improving intensity and angular sensitivity several-fold over traditional surface-plasmon-resonance (SPR) structures and providing an ultra-sensitive platform for biosensing.^143^ In 2022, Wang et al. first demonstrated a significant spin Hall shift in a 100 nm silicon nitride photonic crystal slab, uncovering the enhancement mechanism of PSHE by topological vortices of BICs in momentum space.^144^ In 2023, Wu et al. proposed polarization-dependent q-BICs in a compound grating waveguide structure. By tailoring structural parameters, they introduced a wavelength difference between BICs of TE and TM modes, achieving a remarkable enhancement of the lateral PSHE displacement.^145^ In 2025, Wan et al. optimized the q-BIC metasurface structure, efficiently enhancing the lateral PSHE displacement by combining ultra-high *Q*-factors with a large Fresnel coefficient ratio. This study enables flexible tuning of the incident angle corresponding to the maximum displacement by adjusting the substrate thickness, improving the engineering applicability of the device.^146^

The q-BICs provide a universal and efficient platform for beam shift enhancement, with their core advantages lying in the strong phase modulation and high coupling efficiency enabled by ultra-high *Q*-factors. For GH shift enhancement, displacement magnitude breakthroughs have been achieved in structural configurations encompassing hybrid gratings, photonic crystals, and other forms, with applications in high-sensitivity sensing, optical storage, wavelength-division multiplexing and beyond. For PSHE enhancement, the order-of-magnitude improvement in lateral displacement opens up new possibilities for spintronic applications such as spin filtering and polarization manipulation. Future directions include optimizing designs to boost *Q* factors and displacement amplification, developing integrated miniaturized devices based on this mechanism, exploring multi-field control for dynamically tunable displacements, and advancing practical implementation in integrated spin-optoelectronic devices and ultra-high-precision sensing.

### Non-local wavefront and momentum-space polarization control enabled by BICs

Beyond localized field enhancement and high-*Q* spectral responses, BICs have recently enabled a new class of photonic functionalities based on non-local wavefront and momentum-space polarization control. In contrast to conventional metasurfaces, where wavefront shaping is achieved through spatially varying local phase delays encoded at the level of individual meta-atoms, BIC-based platforms exploit extended, delocalized resonant modes whose radiation properties are governed by global symmetry and momentum-space topology. As a result, the output wavefront and polarization characteristics are determined collectively by the modal structure rather than by pixel-level phase engineering, establishing a fundamentally different paradigm for flat-optics design. Recent studies have demonstrated that BICs and quasi-BICs supported by photonic crystal slabs can sustain long-range phase coherence across large areas, allowing a single resonant mode to generate complex wavefronts, such as focusing, beam steering, and multifunctional beam shaping. This non-local response originates from the Bloch nature of BIC-associated modes, whose far-field radiation is dictated by their dispersion and topological features in reciprocal space.^147^^,^^148^^,^^149^ Compared with traditional local-phase metasurfaces, non-local BIC-based wavefront modulators significantly reduce structural complexity while improving fabrication tolerance and energy efficiency, thereby offering a scalable route toward large-area and multifunctional flat-optical devices. Closely intertwined with non-local wavefront modulation is the control of polarization singularities in momentum space, particularly C points corresponding to purely circular polarization states. In BIC-supporting systems, polarization vortices associated with topological BICs naturally give rise to C points in the surrounding k space, whose positions, charges, and trajectories are intrinsically linked to the symmetry and topology of the underlying resonant modes. By tuning geometric parameters or introducing controlled symmetry breaking, these C points can be deterministically created, displaced, merged, or annihilated, enabling systematic manipulation of circular polarization channels and spin-dependent radiation processes.^150^^,^^151^^,^^152^

Importantly, the regulation of C points via BICs is inherently global and topological in nature. Unlike conventional polarization-control strategies based on local anisotropy or birefringent phase retardation, BIC-enabled C-point engineering relies on momentum-space topology and polarization-vortex conservation laws. This approach provides enhanced robustness against disorder and enables predictable evolution of polarization singularities under continuous parameter variations. Consequently, BIC-based platforms have enabled advanced functionalities, such as chiral wavefront shaping, vector-beam generation, spin-orbit coupling control, and polarization-multiplexed photonic devices. Taken together, non-local wavefront modulation and momentum-space C-point control represent a unified frontier enabled by BIC physics, in which phase, amplitude, and polarization are coherently governed by extended resonant modes and topological constraints. This capability highlights a qualitative advantage of BIC-based photonic systems over conventional resonant and metasurface platforms, reinforcing their emerging role as a foundational framework for next-generation flat optics and multifunctional photonic technologies.^153^^,^^154^

In addition to mainstream applications, BICs show broad potential in molecular sensing, dynamic control, and on-chip integration. Cambiasso et al. first applied BIC to surface-enhanced Raman scattering (SERS). Through the high-*Q* local field, the sensitivity of molecular detection was greatly improved, promoting the development of metal-free enhancement platforms.^110^ The Romano team developed a dual-mode fluorescence and Raman enhancement structure based on all-dielectric BIC metasurfaces, offering a new route for multimodal biological sensing.^111^ Fan et al. exploited BIC tunability to achieve dynamic control of resonance frequency and excited-state lifetime, advancing programmable optical systems.^155^ In 2023, the Li team designed an ultra-narrow-band chiral BIC structure, achieving a highly selective circular dichroism response, which is suitable for precise polarization detection and optical encoding.^156^ Wang et al. developed a high-sensitivity BIC refractive index sensor, significantly reducing the detection limit.^112^ In 2019, Yesilkoy et al. reported in Nature Photonics a BIC-based metasurface biosensor that enables hyperspectral imaging and molecular-level detection, demonstrating prospects for wearable devices and environmental monitoring.^108^ In 2020, the Ndao team first applied BICs to a real time single cell exosome monitoring system, providing a high sensitivity optical tool for single cell biology research.^113^ In 2022 Qin et al. proposed BIC-induced topological optical forces for reversible trapping and three-dimensional asymmetric manipulation of nanoparticles, demonstrating the potential of BICs in optically driven micro- and nano-manipulation.^157^ In 2025 Yang et al. reviewed applications of BICs in optical sorting, showing that they enable sub-nanometer-resolution and high-selectivity particle manipulation, thereby offering a new route for efficient sorting of biological particles, viruses, and exosomes.^158^

Overall, BICs have evolved from a high-*Q* physical phenomenon into a photonic platform enabling multi-physics coupling, dynamic tuning, and functional integration. In lasers, nonlinear optics, chiral control, and terahertz technology, researchers have moved from principle verification to functional application by continually pushing the boundaries of materials and structures. However, current work remains largely confined to single function and static designs. The lack of a unified theory for large-scale design, stability analysis in complex environments, and cross-frequency band system integration strategies are still key problems hindering its industrialization. Future research should address: (1) heterogeneous integration of BICs with two-dimensional materials, quantum dots and chiral media; (2) dynamic-control methods for high-dimensional multimode BICs; (3) mechanisms that ensure BIC stability against fabrication errors and thermal disturbances; (4) BIC-chip design methodologies based on collaborative simulation and intelligent inverse design. Through the previous explorations, BIC is expected to upgrade from a special physical phenomenon in nanophotonics to a core technical foundation of a multifunctional integrated photonics platform, promoting the development of photonic devices toward high performance, intelligence, and systematization.

## Conclusion

### Emerging opportunities and challenges for BIC-based photonic platforms

Despite the rapid progress of BICs across diverse photonic platforms, translating their unique physical properties into scalable and multifunctional systems introduces a series of interconnected challenges. Rather than reiterating application-level performance metrics, this section focuses on the underlying physical and engineering constraints that currently limit the practical deployment of BIC-based platforms. From a materials perspective, extending BIC concepts beyond conventional low-loss dielectrics introduces both new opportunities and intrinsic trade-offs. Active and functional materials—such as phase-change media, two-dimensional semiconductors, and ferroelectrics—enable dynamic modulation, memory effects, and multi-physical coupling of BIC states. However, these materials also introduce additional absorption, dispersion, and thermal instability, which can strongly perturb the delicate interference or symmetry conditions required for BIC formation. Developing co-design strategies that jointly optimize material response and modal robustness therefore remains a critical challenge.

At the architectural level, many emerging BIC-enabled functionalities rely on global modal coherence rather than localized resonant control. While this feature underpins non-local wavefront shaping and polarization singularity engineering, it also raises fundamental questions regarding scalability and controllability. In particular, maintaining coherent collective modes under intentional symmetry breaking, dynamic modulation, or partial disorder requires a deeper understanding of how BICs evolve in high-dimensional parameter spaces. Achieving deterministic and reversible control over such evolution is essential for programmable and adaptive photonic architectures.

Fabrication tolerance constitutes another central challenge. Although topological and momentum-space descriptions of BICs suggest intrinsic robustness, experimental demonstrations of fabrication-invariant performance remain limited. Concepts such as merged BICs and topological charge conservation provide promising theoretical routes toward tolerance-aware design, yet systematic experimental validation and quantitative tolerance metrics are still largely lacking. Finally, system-level integration presents challenges that extend beyond individual device performance. Coupling BIC-enabled elements with on-chip sources, detectors, and control electronics introduces additional constraints associated with thermal management, electrical tuning, and heterogeneous integration. Addressing these issues requires unified design frameworks that bridge BIC physics with photonic circuit engineering and system-level optimization.

Taken together, these challenges indicate that advancing BIC-based photonics beyond proof-of-concept demonstrations requires a deeper understanding of material-mode interactions, architectural control of global coherence, and tolerance-aware design strategies. Addressing material, architectural, fabrication, and integration constraints therefore remains an open problem and a central focus of ongoing research in the field.

### Conclusion and outlook

Overall, although BICs have enabled a broad spectrum of high-impact applications—from ultra-high-*Q* resonators and low-threshold lasers to nonlinear enhancement, polarization control, and non-local wavefront modulation—their practical deployment still faces several shared limitations that transcend individual application categories. Across different platforms, these challenges mainly manifest as sensitivity to fabrication imperfections, trade-offs between field confinement and external coupling, limited dynamic tunability, and difficulties in scaling from single-device demonstrations to system-level implementations.

A first and widely encountered limitation lies in fabrication tolerance. Ideal BICs often rely on strict symmetry or precise interference conditions, making their performance vulnerable to structural disorder, particularly in applications such as BIC lasers and ultra-narrowband sensors. In response, increasing attention has been directed toward quasi-BIC and topology-assisted design strategies, including controlled symmetry breaking, merged BIC configurations, and momentum-space topological charge conservation. These approaches preserve high-*Q* behavior while ensuring that performance degrades gradually rather than catastrophically under realistic fabrication conditions. A second challenge concerns the balance between strong field confinement and efficient energy extraction. While ideal BICs offer infinite *Q* in theory, their complete decoupling from radiation channels limits practical usability in applications requiring efficient output. This trade-off has motivated the deliberate use of quasi-BICs, where radiative coupling is engineered to an optimal level, enabling simultaneous field enhancement and usable bandwidth in nonlinear metasurfaces and photonic crystal slabs.

Dynamic control and reconfigurability constitute another major bottleneck, as many BIC-based devices remain inherently static. Emerging solutions based on phase-change materials, thermo-optic or electro-optic tuning layers, and hybrid material platforms have demonstrated reversible switching between BIC and quasi-BIC states, as well as tunable wavefront and polarization control. Finally, system-level integration remains an open challenge. While individual BIC-enabled components exhibit outstanding performance, their integration into large-area photonic circuits requires unified design principles that address loss management, thermal effects, and scalability. In this context, non-local and topology-driven BIC platforms are particularly promising, as their global modal nature facilitates collective control across extended structures.

In summary, the future impact of BIC-based photonics will depend not only on discovering new resonant effects, but more critically on addressing shared limitations through fabrication-tolerant design, controlled radiative coupling, dynamic reconfigurability, and scalable integration strategies. By consolidating these response actions across different applications, BICs are poised to evolve from isolated high-*Q* phenomena into a robust and versatile platform for next-generation multifunctional photonic systems. As a core concept in nanophotonics, BICs exhibit ultra-high *Q* factors and strong field localization, with unique advantages in nonlinear enhancement and chiral manipulation. This review has systematically summarized the physical origin, classification, theoretical frameworks, and cutting-edge applications of BICs in periodic optical systems. However, the translation of BICs from theoretical constructs to practical devices still faces challenges, including sensitivity to fabrication precision, limited dynamic tuning approaches, complex multimodal coupling, and incomplete exploration of non-Hermitian and topological effects.

Looking forward, breakthroughs in BIC research are expected along several key directions: (1) expansion of material platforms toward low-loss, high-refractive-index, and CMOS-compatible systems, such as SiN, Ge, TiO_2_, perovskites, and two-dimensional materials; (2) innovation in structural design through inverse design and machine-learning-assisted optimization, enabling multimodal, broadband, and angle- or polarization-insensitive BICs; (3) development of dynamic tuning mechanisms based on phase-change materials, liquid crystals, and low-dimensional materials for real-time reconfigurability; and (4) integration of topological and non-Hermitian concepts, including the exploration of BICs near higher-order topological states and exceptional points, to realize robust and directionally radiating photonic states. With continuous advances in nanomanufacturing, materials synthesis, and intelligent design methodologies, BICs are expected to evolve from high-*Q* resonators into core building blocks of multifunctional integrated photonic platforms, playing a pivotal role in next-generation systems such as on-chip light sources, quantum information processing, single-molecule biological detection, and high-sensitivity intelligent sensing.

## Acknowledgments

This work was supported by 10.13039/501100001809National Natural Science Foundation of China (no. 62475293 and 62105376), Science and Technology Development Fund from Macao (Special Administrative Region) SAR (FDCT) (0002/2024/TFP), 10.13039/501100021171Guangdong Basic and Applied Basic Research Foundation (no. 2024A1515011727), Fund of State Key Laboratory of Photonics and Communications, P.R. China under grant no. 2025QZKF08, Guangdong Zhujiang Project (grant nos. 2021ZT09X070 and 2021QN02X488).

## Declaration of interests

The authors declare no competing interests.
